# Telephone interventions for adherence to colpocytological examination^1^

**DOI:** 10.1590/1518-8345.1683.2844

**Published:** 2017-02-06

**Authors:** Thais Marques Lima, Ana Izabel Oliveira Nicolau, Francisco Herlânio Costa Carvalho, Camila Teixeira Moreira Vasconcelos, Priscila de Souza Aquino, Ana Karina Bezerra Pinheiro

**Affiliations:** 2 PhD, Assistant Professor, Centro Universitário Estácio do Ceará, Fortaleza, CE, Brazil.; 3 PhD, Adjunct Professor, Departamento de Medicina, Universidade Federal do Ceará, Fortaleza, CE, Brazil.; 4 PhD, Adjunct Professor, Departamento de Enfermagem, Universidade Federal do Ceará, Fortaleza, CE, Brazil.

**Keywords:** Uterine Cervical Neoplasms, Intervention Studies, Telephone, Nursing

## Abstract

**Objective:**

to test the effects of behavioral and educational intervention by telephone on adherence of women with inappropriate periodicity to colpocytological examination.

**Method:**

quasi-experimental study with a sample of 524 women, selected with the following inclusion criteria: be aged between 25 and 64 years, have initiated sexual activity, have inappropriate periodicity of examination and have mobile or landline phone. The women were divided into two groups for application of behavioral and educational intervention by telephone. It was used an intervention script according to the principles of Motivational Interviewing.

**Results:**

on comparing the results before and after the behavioral and educational interventions, it was found that there was a statistically significant change (p = 0.0283) with increase of knowledge of women who participated in the educational intervention. There was no change in the attitude of women of any of the groups and there was an increase of adherence to colpocytological examination in both groups (p < 0.0001), with greater adherence of women participating in the behavioral group (66.8%).

**Conclusion:**

the behavioral and educational interventions by phone were effective in the adherence of women to colpocytological examination, representing important strategies for permanent health education and promotion of care for the prevention of cervical cancer.

## Introduction

Cervical cancer (CC) is the third malignant neoplasia with the highest incidence in the world and the fourth leading cause of death from cancer in women, which is directly related to the degree of development of some countries, mainly affecting women with low socioeconomic status and less access to screening actions^1^.

The incidence of this cancer is higher in the age group between 30 and 39 years and its control is a challenge to public health because of the financial and social damage associated with the disease, considering that, when they are sick, women occupy hospital beds, leave the labor market and are deprived of family life^2^.

In order to reduce the morbidity and mortality rates for this cancer, the Brazilian Ministry of Health adopted as standard the recommendation of the World Health Organization. It proposes the cytological examination of the cervix every three years, after two consecutive negative annual exams in women aged between 25 and 64 years, or who, at that moment, have initiated sexual activity, in addition to improving the adherence to screening strategies^3^.

It is known that women who are at increased risk of developing this cancer are not being reached by screening programs, due to the fact that many of these women attended the healthcare units to undergo the examination for reasons unrelated to cancer prevention. In this way, the lack of adherence to periodicity of the colpocytological examination is considered the main limiting factor for detection of CC^4^. 

Among the most important factors related to non-adherence to periodicity of the colpocytological examination are the low socioeconomic status, low education, lack of a partner, fear of doing it and/or a positive test result for cancer and constraint; smoking; women’s lack of time; difficult access to the health service and lack of knowledge about the colpocytological examination^5^.

In this way, in addition to the existing socioeconomic inequalities, there is the fact that cervical screening is mainly opportunistic, serving women who are at that moment in the healthcare unit, without greater efforts to reach the population considered to be at higher risk. Most healthcare services are restricted to younger women seeking primary healthcare, prenatal care or family planning, in which the examination is performed aiming at treating or preventing other health problems^6^.

In this context, the nurse, as a health professional, has a fundamental role in the development and practice of interventions aimed at changing this reality, considering that the focus of this profession should be the healthcare in general. Interventions should take into account the individuals and subjectivities of each region, and must be performed differently, considering the individuality and the way of living of each woman^5^.

Therefore, the telephone intervention arises as a potential tool for the integral health care and means an expansion of the action in health, representing an advance in the traditional nursing care, emerging as potential strategy in the holistic care^7^. In telephone counseling, a standardized protocol must be used to identify the barriers that prevent the individuals from having a good attitude in relation to their health, and provide information to help them to address and overcome these barriers^8^. 

Given the above, there is a need to introduce new behavioral and educational interventions that increase the adherence to colpocytological examination and aim at women’s empowerment. Thus, it has been questioned: What will be the effects of an educational intervention and a behavioral intervention, by telephone, on the adherence to colpocytological examination by women who have inappropriate periodicity? The aim of this study is to test the effects of the behavioral and educational interventions by telephone on the adherence of women with inappropriate periodicity to colpocytological examination.

Such studies are important because they aim to propose and assess the use of interventions to minimize the absenteeism rates to the regular consultations for colpocytological examination and, thus, improve the healthcare of women; reduce the unnecessary health system expenditures and stimulate nurses to undertake new interventions, which are essential for success in the control of CC.

## Method

This is a quasi-experimental study with pre-test and post-test design, developed in the city of Fortaleza, at the Family Development Center (CEDEFAM), from June to December 2014.

The study population consisted of women who underwent examination for prevention of cervical cancer at that healthcare unit, and did not show the periodicity to examination in accordance with the recommendations of the Ministry of Health, i.e., they did not undergo the annual colpocytological examination or any examination in the last three years, after two consecutive negative tests^3^. The sample selection met the following inclusion criteria: women aged between 25 and 64 years, who have initiated sexual activity, with inappropriate periodicity of examination and who had their mobile or landline phones numbers registered in the medical records. To classify them as “inappropriate periodicity”, the amount of previous examinations was also assessed. 

The sample size calculation was performed using the formula for comparative studies of groups and the following values were adopted: Zα = 95%, Zβ = 80%, *p* = 18.6%, d = 10%. Thus, by substituting the values, it was found that 239 women would be needed for each group. However, a 10% safety percentage for possible telephone losses was added, with 262 women for each group, totalling 524 women.

The women composing the sample were randomly allocated into two groups:

- Group 1. educational telephone intervention: an educational telephone intervention and the scheduling of the colpocytological examination was offered to the women. The intervention was guided by the technology created in a previous study^9^ and by the guidelines for cervical cancer control of the Ministry of Health^3^, in accordance with the principles of Motivational Interviewing (MI)^10^. During the phone call, demographic data and information on knowledge, attitude and practice of women on the prevention of cervical cancer were also collected through application of the KAP survey.

- Group 2. behavioral telephone intervention: a behavioral telephone intervention (telephone reminder) and the scheduling of the colpocytological examination was offered to the women. During the phone call, demographic data and information on knowledge, attitude and practice of women on the prevention of cervical cancer were also collected through application of the KAP survey.

The study design involved the random assignment of subjects to groups. By means of randomization, or random selection for groups, all participants had an equal chance of being included in either group. It was predicted from the beginning of the study that the randomly assigned groups were similar, in general, in relation to the infinite number of biological, psychological and social features^11^ ^).^ The random selection was performed by means of a table created by using a random allocation software. Each number on the lists of the two groups (educational and behavioral) was inserted into an opaque envelope, which was numbered and sealed. Individuals unaware of the study performed these steps. 

In this study, women who were part of the sample were blinded with respect to the group to which they belonged. Besides them, two professionals were chosen - one of them performed the behavioral telephone interventions and the other performed the educational telephone interventions. These trained professionals were also blinded, because they were not aware of the goals of the study and they did not know the schedule and the existence of groups. 

The educational telephone intervention was divided into two phases: the first phase consisted of the introduction of the professional, research on the examination performed in other healthcare services and questions about the intention to participate in the study; if the answer was affirmative, the second phase was started. 

In the second phase, information on the sociodemographic characteristics and knowledge and attitude of women on the prevention of CC were collected. Subsequently, the intervention started, which addressed: a brief explanation on CC and its risks, the purpose of the colpocytological examination, the importance of the periodicity of examination, pre-examination care and return to receive the result of the colpocytological examination^3^ ^,^ ^9^. 

The evoke-provide-evoke model was used, supported by the MI, because it involves a collaborative mindset, proper for interventions aiming at behavior changes. This model seeks not only to transfer information, but also to insert the patients into the context and enable them to make their own decision about the change^10^.

The two phases were carried out in a single telephone call, lasting about fifteen minutes, because this is the recommended time for intervention^12^. Finally, the examination was scheduled according to the availability of women to attend the healthcare unit.

The behavioral intervention used in this study aimed to serve as a reminder to the colpocytological examination. This telephone intervention was divided into two phases - the first phase consisted of the introduction of the professional, research on the examination performed in other healthcare services and questions about the intention to participate in the study; if the answer was affirmative, the second phase was started. 

In the second phase, information on the sociodemographic characteristics and knowledge and attitude of women in relation to prevention of CC were collected. Subsequently, they received a reminder for the examination without any information about it. In addition, the examination was scheduled according to the availability of women to attend the healthcare unit. These two phases were carried out in a single telephone call, lasting about five minutes.

The second phase of data collection was carried out on the day of consultation, which was scheduled to perform the colpocytological examination. The KAP survey was applied again to women who attended the healthcare unit, in order to compare the effects of the intervention on their knowledge and attitude in relation to the examination. Next, colpocytological examination was performed and another day was scheduled to receive the results of the examination (from 30 to 40 days after examination). 

Data were compiled and analyzed using the *Statistical Package for the Social Sciences* software, version 20.0. Continuous variables were expressed as mean ± Standard Deviation, with a 95% confidence interval (CI), and categorical variables as frequencies and percentages. To assess the knowledge, attitude and practice of women regarding the colpocytological examination before and after interventions, it was used Person’s chi-square test and McNemar’s test. To assess the existence of factors related to knowledge, attitude and inappropriate practices on the examination, it was used Person’s chi-square test and *odds ratio* (OR), with a 95% confidence interval. 

In the assessment of the existence of factors related to non-performance of the colpocytological examination, it was also used the Pearson’s chi-square and OR. A *p*-value ≤ 0.05 was considered statistically significant.

Compliance with standards for research involving humans was ensured by the Resolution 466/2012 of the National Health Council of Brazil^13^. Initially, it was requested authorization from the Coordination of CEDEFAM for conducting this study. Next, the study was submitted to ethical approval by the Brazil Platform, which approved it under opinion number 700.006.

## Results

It was observed that the average age was 38.4 years, considering the minimum and maximum ages recommended by the MOH (25-64 years). Women showed an average of 9.4 years of study, prevailing complete high school level (32.6%). 

Regarding the onset of sexual life (OSL), the average age was 17.3 years, with 11 years as minimum age and 32 years as maximum age. Most participants reported having a partner (53.4%), not work outside the home (52.9%) and reside near to the healthcare unit where the examination was performed (88.7%).

When asked about the date of the last colpocytological examination, it was observed an average of 31.7 months, ranging from 13 to 122 months.

The motives that make women not to undergo the colpocytological examination periodically were also questioned. Many of the study participants mentioned the difficulty in scheduling the examination in the healthcare unit as the major impediment to attend with the appropriated periodicity (40.8%). It is noteworthy, however, the presence of factors such as personal negligence (23.7%), forgetfulness about the period of undergoing the examination (12.0%), lack of time (10.5%) and even the lack of interest (5.3%).

In assessing the knowledge about the colpocytological examination and attitude in relation to its performance before the intervention, it was found that most women had inappropriate knowledge (68.7%) and attitude (61.1%). It is emphasized that all women were classified as “inappropriate practice of examination”.

Assessments were performed before and after each intervention to evaluate the effect of interventions on the knowledge, attitude and practice on the adherence to examination, as shown in the tables that follow. It is emphasized that, of the total of 524 women, 198 (37.7%) did not show up to undergo the colpocytological examination.

Table 1 shows the assessment of knowledge about the colpocytological examination between the behavioral and educational groups assessed with the use of KAP survey.

**Table 1:** Assessment of knowledge before and after application of the interventions. Fortaleza, CE, Brazil, 2014

Intervention		Groups			P^1^*	
		Behavioural		Educational		
		n	%	n	%	
Before						0.451
	Inappropriate	176	67.2	184	70.2%	
	Appropriate	86	32.8	78	29.8%	
After						0.497
	Inappropriate	113	64.6	92	60.9%	
	Appropriate	62	35.4	59	39.1%	
p^2^ ^)†^		0.056		0.0283		

*P^1^ ^)^ = Chi-square test; ^†^p^2^ ^)^ = McNemar test

In both groups there was a prevalence of inappropriate knowledge before and after intervention, being higher than 60.9%. However, it is observed that there was a statistically significant increase of knowledge in the educational group (*p*² = 0.0283) when this group was analyzed before (29.8%) and after (39.1%) intervention, and almost 10% of women acquired appropriate knowledge about the examination. Regarding the behavioral group, it was observed a small change in the adequacy of knowledge in this group before (32.8%) and after (35.4%) intervention. Nevertheless, this change leads us to believe that when examination was scheduled through a reminder intervention, women became interested to know more about the examination, acquiring knowledge by their own means.

Table 2 shows the assessment of women’s attitude in relation to the examination before and after interventions. 

**Table 2:** Assessment of attitude before and after application of the interventions. Fortaleza, CE, Brazil, 2014

Intervention		Groups			P^1^*	
		Behavioural		Educational		
		n	%	n	%	
Before						0.857
	Inappropriate	159	60.7	161	61.5	
	Appropriate	103	39.3	101	38.5	
After						0.720
	Inappropriate	110	62.9	92	60.9	
	Appropriate	65	37.1	59	39.1	
p^2^ ^)†^		0.056		0.0283		

*P^1^ ^)^ = Chi-square test; ^†^p^2^ ^)^ = McNemar test

There was a prevalence higher than 60.9% of the inappropriate attitude before and after intervention in both groups, but this association was not statistically significant in the behavioral group (*p* = 0.631) and in the educational group (*p* = 0.681). In the educational group, there was a slight reduction in the inadequacy of attitude before (61.5%) and after (60.9%) intervention. In the behavioral group, there was a small increase of inappropriate attitudes, when it was compared before (60.7%) and after (62.9%) intervention.

Table 3 shows the assessment of the practice in relation to the adherence to colpocytological examination in the groups. Before the interventions, all women in the study were included in the inappropriate practice group, but in comparing the groups after the interventions, it was found a statistically significant relationship (*p* < 0.0001) for the two groups, showing that interventions were effective in raising the attendance of women to the healthcare services for examination.

**Table 3:** Assessment of adherence before and after application of the interventions. Fortaleza, CE, Brazil, 2014

Intervention		Groups				P^1^*
		Behavioural		Educational		
		n	%	n	%	
Before						1.000
	Inappropriate	262	100.0	262	100.0	
	Appropriate					
After						0.031
	Inappropriate	87	33.2	111	42.3	
	Appropriate	175	66.8	151	57.5	
p^2^ ^)†^		<0.0001		<0.0001		

*P^1^ ^)^ = Chi-square test; ^†^p^2^ ^)^ = McNemar test

It was also noted that there was a higher percentage of adherence to examination among women in the behavioral group (66.8%), in comparison with the educational group (57.7%), showing that most of the women who participated in the educational intervention did not show up for examination (42.3%), even after they have increased their knowledge about the importance of undergoing it.

## Discussion

The average age found in this study corroborates a study of 772 women aged over 18 years, carried out in the city of Rio Branco, Acre, showing an average age of 36.6 years, with a higher prevalence of undergoing the colpocytological examination among women aged between 25 and 35 years (86.4%). According to the authors of this study, the end of child-bearing age influences in reducing the appointments for gynecological consultations or their demand in the healthcare units, leading to decrease of prevention practices in the age period in which the incidence and severity of cancer are higher^14^.

Added to the above, non-adherence to appropriate periodicity to the colpocytological examination is related to some social factors such as be part of the younger age group, be single and have low education or low socioeconomic status^15^. 

There was a large time gap since the last colpocytological examination, with an average of 31.7 months. The periodical examination is essential, since the late diagnosis of CC can cause not only physical injury, but also emotional and psychosocial problems^16^.

Women undergoing examination at a periodicity exceeding three years also have higher proportion of no returning to receive the final results of examination, and they do not show the results to a professional, featuring discontinuity of this health care^17^.

When asked about the reason for not undergoing examination, many women mentionated the difficulty in scheduling the examination, carelessness, lack of time, among other. A study of 12 women diagnosed with CC found that there are problems related to the women, healthcare services and professionals, what makes it difficult the examination as recommended. This study found that factors such as shame, carelessness, fear of going to the doctor, absence of symptoms and lack of time are individual components that interfere with the practice of examination. Moreover, since they affected the demand for examination in the healthcare services, these reasons denote lack of knowledge about the importance and necessity of periodical examination^16^.

Undergoing examination at an inappropriate periodicity can be associated with inappropriate knowledge and attitude, as observed in this study. A study of 30 women in the state of Minas Gerais found that only 40% had appropriate knowledge about the colpocytological examination and 83.3% did not know what was HPV or reported - erroneously - that it was the “same as” AIDS, cancer, some kind of bacteria and so on^18^.

In assessing the knowledge before and after intervention, it was found that in both groups there was a prevalence of inappropriate knowledge, exceeding 60.9%. Therefore, it is necessary to improve the knowledge and awareness of women about prevention of cervical cancer, and educate the population so that the demand for colpocytological examination meets the screening goals, also reaching the most excluded women^6^. 

Regarding the attitude towards the examination, there was a prevalence higher than 60.9% of inappropriate attitude before and after intervention in both groups. It is emphasized that none of the interventions proposed have improved women’s attitude. This may be related to the culture of seeking the healthcare services to undergo the examination only when there are symptoms, or to check the health status, not associating the prevention of CC as the main reason for examination. 

Promoting the development of personal skills and attitudes favorable to health at all stages is among the most import fields of health promotion, considering that they provide opportunities for the development of the health potential. To this end, it is essential the dissemination of information on health education, which should take place in all collective spaces. Friendly environments, access to information, skills to live better, just as opportunities to make healthier choices, are among the main empowerment elements^19^.

Regarding the adherence to examination, by comparing the groups after the intervention, it was found a statistically significant relationship (*p* < 0.0001) for the two groups, showing that interventions were effective. It is worth mentioning the greater attendance by women belonging to the behavioral group. However, it is known that behavioral interventions appear to have more elusive effect when compared to cognitive interventions, leading to the need to use a combination of interventions to achieve a better efficacy^17^. Thus, the women in this study showed a higher increase in knowledge and lower adherence rate in the educational group. Therefore, it is recommended to carry out a further study to assess the effectiveness of adherence over time in both groups after one year, confirming the permanent modification about the practice of colpocytological examination.

In this way, performing telephone interventions is a resource associated with the nursing practice, which can produce significant changes in the health of the population, highlighting the importance of technical and clinical knowledge for the interventions by the professional. Moreover, the use of technology for healthcare development requires trained professionals to promote the convergence between human development and technological knowledge, aiming at the desired goals^20^.

Investing in prevention practices and awareness of the population is less costly than the curative treatment of various types of diseases, especially in the public health system, by reducing the costs of hospitalization, surgeries and treatments^19^.

In a case study of a chemically dependent patient, the use of MI by telephone enabled the codependency to be reduced, and modified the observed permissive behavior. Moreover, the authors noticed the expansion of self-perception of the patient on his personal needs and limits with regard to chemical dependency. Thus, it is emphasized that an intervention focused on motivational approaches and in MI represents a helping strategy^21^.

The educational intervention based on principles of MI was able to arouse the interest of participants to change their demotivation in relation to self-care and caused changes that favored the prevention of diseases, in this case, the undergoing of colpocytological examination. It is emphasized that no person is completely unmotivated and the motivation to change is quite malleable and formed especially within the context of relationships. Great results are achieved when patients are interested and show an active role, making the intervention more effective and with a lasting influence on the patient’s health^10^.

## Conclusion

It was found, in the view of the observed changes, that the different telephone interventions were effective in promoting the adherence of women to colpocytological examination. For this reason, it is considered relevant the permanent healthcare education, with actions that aim at promoting the prevention of CC and are comprehensive, prioritizing interventions for the screening of both the asymptomatic and symptomatic women, besides ensuring access to methods for proper diagnosis and treatment.

It is recommended conducting future studies to assess the long-term effectiveness of the educational and behavioral interventions on adherence to colpocytological examination, in order to check, after a year, if women remain seeking the healthcare service to undergo the examination within the recommended periodicity.
